# Predictors of neonatal mortality in Assosa zone, Western Ethiopia: a matched case control study

**DOI:** 10.1186/s12884-019-2243-5

**Published:** 2019-03-29

**Authors:** Fillmon Kidus, Kifle Woldemichael, Desta Hiko

**Affiliations:** 1 Department of Disease Prevention and Health Promotion, Benshangul Gumuze Regional Health Bureau, Assosa, Ethiopia; 2 Department of Epidemiology, Colleage of Public Health and Medical Science, Jimma University, Jimma, Ethiopia

**Keywords:** Neonatal mortality, Case control, Predictors

## Abstract

**Background:**

Benshangul Gumuze region is one of the regional states in Ethiopia, with highest rate of neonatal mortality rate. The trend increased at alarming rate from 42/1000 live birth in 2005 to 62/ 1000 live birth in 2011. Hence, identifying predictors of neonatal death and implement evidence based interventions at community level is crucial to reduce the mortality. Therefore, the purpose of this study was to identify predictors of neonatal mortality in Assosa zone, Western Ethiopia.

**Methods:**

A community based matched case control study was conducted from February 1, until December 30, 2013. The study included 114 cases who died during the first 28 completed days after birth from September 1, 2010 till September 1, 2013. For each case, one alive control matched approximately by the same date of birth (−/+ 2 days) was identified from the preliminary data collected. Finally, multivariate conditional logistic regression analysis was performed; and goodness of fit of the final model was tested using likely hood ratio test. All analysis was done using EPI Info version 7 and SPSS version 16 statistical softwares.

**Results:**

Model households in health extension packages [AmOR = 0.32; 95%CI:0.12–0.86], age at first pregnancy < 20 years old [AmOR = 4.3;95%CI: 1.13–16.27],pregnancy complication [AmOR = 4.59; 95%CI: 1.53–13.78], delivery complication [AmOR = 2.80; 95%CI: 1.06–7.39], antenatal care visit [AmOR = 0.34;95%CI: 0.12–0.94], primipara mothers [AmOR = 3.37; 95%CI:1.05–10.78], small size neonate at birth [AmOR = 3.40: 95%CI: 1.05–11.55], gestational age < 37 weeks [AmOR = 4.35;95%CI:1.16–16.28], and home delivery [AmOR = 2.84; 95%CI:1.07–7.55] were found statistically significantly associated with neonatal mortality.

**Conclusions:**

Model households in health extension package and antenatal care visit were associated with reducing risk of neonatal mortality. However, age at first pregnancy < 20 years old, primipara mothers, pregnancy complication, delivery complication, small size neonates, gestational age < 37 weeks, and home delivery were associated with increasing risk of neonatal death. Therefore, promotion of model household in health extension package, anti natal care visit, institutional delivery, family planning to prevent early age pregnancy; and improve access to basic emergency obstetric care and intensive newborn care centers are effective interventions to reduce risk of neonatal mortality at community level.

**Electronic supplementary material:**

The online version of this article (10.1186/s12884-019-2243-5) contains supplementary material, which is available to authorized users.

## Background

According to World Health Organization definition, neonatal mortality (NM) is death among live births during the first 28 completed days of life [1]. This may subdivided into early neonatal death, which occur during the first seven days of life (0–6) and late neonatal death occur after the 7th day but before the 28th completed day of life (7–27) [1]. Neonatal period carries one of the highest risks of death in any 4-week period in the human lifespan [2].

Six million nine hundred thousand children died before reaching their fifth birthday globally in 2011; about 43% of this deaths was NM [3]. According to global estimate report, NM is highest in low-income countries and consistently decline with increasing regional income [4]. The heaviest burden is in South Asia and Sub-Saharan Africa; both have highest neonatal mortality rates (NMR) among all regions [4]. The countries in Sub-Saharan Africa (with some exceptions) have made little progress in decreasing such deaths in the past 10–15 years [3, 5].

According to 2011 Ethiopian demographic and health survey (EDHS) report, national NMR in 2000, 2005 and 2011 were 49, 39 and 37 per 1000 live birth respectively [6, 7].

Benshangul Gumuze region is one of the regional states in Ethiopia with highest rate of NM; it was 62 per 1000 live birth in 2011 [7]. The rate was increased at alarm rate from 44 in 2005 to 62 per 1000 live birth in 2011 [6, 7]. Assosa zone is one of the largest populated zone in the region with low coverage of maternal and child health services; such as, institutional delivery, model household in health extension packages, and antenatal care visit [8]. Thus, it might contribute in large proportion to the regional neonatal death profile.

**Fig. 1:**
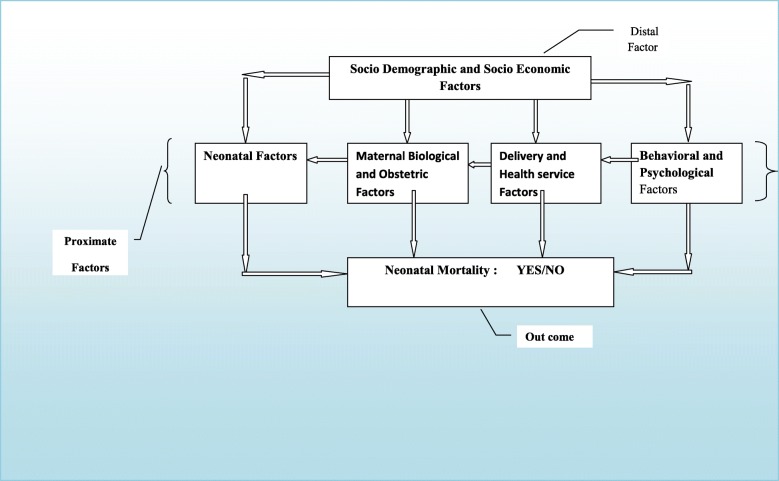
Conceptual framework to study predicators of neonatal mortality, in Assosa zone, Western Ethiopia, 2013

The conceptual framework of this study was adapted from Mosley and Chin framework for the study of child survival in developing countries [9]. It has two major categories: distal and proximate factors. The distal factors were socio-demographic and economic characteristics. The proximate factors were leveled in to four subcategories: maternal biological and obstetric factors, neonatal factors, delivery and health system factors, and behavioral and psychosocial factors (Fig. 1).

Socio demographic and economic factors [10–20], maternal biological and obstetric factors such us: early age 1st pregnancy [17–20], delivery and pregnancy complications [18, 21–24], antenatal care (ANC) visit [18, 19, 21, 24], parity [21, 23], and birth space < 24 months [18, 24, 25] were reported having statistical significant association with NM. In line with this, neonatal factors, like size at birth [21, 26–28], gestational age (GA) < 37 weeks [11, 13, 26, 27], delivery and health system related factors [16, 18, 21, 30–34], and behavioral and psychosocial factors [35, 36] were also shown statistically significantly associated with risk of NM.

In Ethiopia, there were few studies conducted at community level. However, they were only confined to demographic study areas [37, 38]. Moreover, one-size fits all approach is unlikely to work, because the effect of interventions must depend on the local cause-of-death profile and the health system platforms available there. Therefore, the main purpose of this study was to identify predictors of NM. Subsequently, it will add an immense value for efforts to reduce neonatal death in the region, by utilizing the findings of the study for planning and implementation of effective interventions at community level.

## Methods

### Study setting, design and sampling

A matched case control study was conducted in Assosa zone, Western Ethiopia from February 1 until December 30, 2013. Assosa zone is located 667 km to West of Addis Ababa. It has a population of 342,287; male 188,258 and female 154,029 (2007 census projected). The zone has 7 woredas (districts) and 72 kebeles (villages). The study population was sample of neonates who died during the first 28 completed days after birth and sample of neonates who survived the first 28 completed days after birth, from September 1, 2010 until September 1, 2013. Cases were neonates (index birth) who died during the first 28 completed days after birth and controls were neonates (index birth) who survived the first 28 completed days after birth and alive during data collection. Neonates, match the definition of case and control were eligible for the study. However, neonates born outside Assosa zone and neonates mothers who were sick or unable to communicate were excluded from the study.

**Fig. 2:**
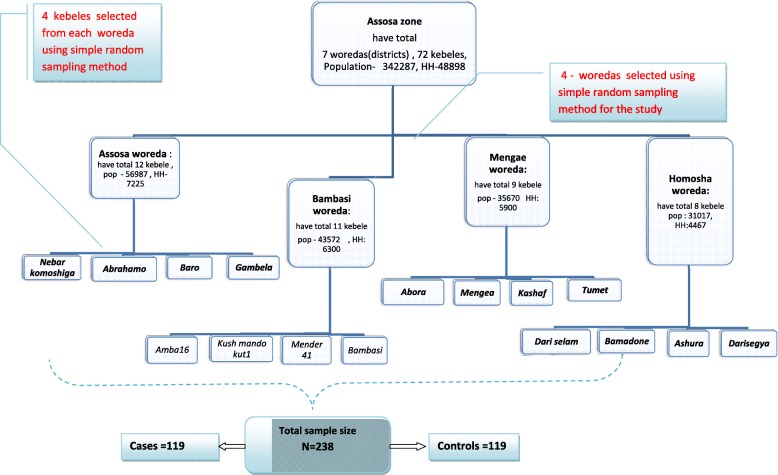
Schematic presentation of sampling technique to study predicators of neonatal mortality in Assosa zone, Western Ethiopia, 2013

Sample size of the study was determined by PS software version 3.0.43 power and sample size calculation for matched case control study. Different predictors were considered to determine the sample size. Thus, preterm birth was chosen, as it gave large sample size using parameters of 95% confidence interval (CI), 80% power, proportion of preterm birth among cases (P_1_) was 48.5%, and proportion of preterm birth among controls (P_0_) was 8.2% [39]. The minimum detectable odds ratio was 3.5 and ratio of case to control (m) 1:1. Correlation coefficient (r) for exposure between matched case and control was unknown; hence, 0.2-phi coefficient was taken on the assumption of dependency [40]. Adding 5% contingency for non-response, the total sample size required for the study was 238(119 cases and 119 controls). With regard to sampling techniques, from the total seven woredas (districts) of the zone, four districts were selected first, then from each four districts, four kebeles (villages) were chosen. Simple random sampling method was used to choose both the districts and villages. As result, totally 16 villages were included in the study to get sufficient sample of cases (Fig. 2).

Preliminary data was collected from the 16 villages’ health posts child health registration book, ahead of the actual study. Information was collected on the following variables: date of birth, date of death, household identification, place of birth and type of birth (singleton or multiple). Then, all live birth neonates who survived the first 28 completed days after birth and all live birth neonates who died during the first 28 completed days after birth from September 1, 2010 till September 1, 2013 were indentified. From the preliminary data result, 139 singleton cases were identified and eight cases were excluded based on the exclusion criteria listed (six cases were born outside Assosa zone and two mothers of cases were unable to communicate). Afterward, 131 eligible cases were identified and sampling frame was prepared. However, due to constraint of resource, only the required samples of 119 cases were selected using simple random sampling method. Finally, for each identified cases, one alive control was matched approximately by the same date of birth (−/+ 2 days) from the preliminary data collected.

### Measurements

Structured questionnaire was prepared in English by reviewing different literatures and adapting World Health Organization verbal autopsy to local context. Then, it was translated to Amharic and checked for consistency by back translation to English by different individuals. The questionnaire had five parts: socio demographic and economic characteristic, maternal biological and obstetric factors, neonatal factors, behavioral and psychosocial characteristics, and health service and delivery related factors.

Model households in health extension package was defined when the households were attended training on 16 health packages (personal hygiene, control of insect and rodents, healthy home environment, food hygiene and safety, water supply and safety, solid and liquid waste, safe excreta disposal, TB and HIV control, malaria prevention and control, first aid and emergency measure, maternal and child health, family planning, immunization, adolescent reproductive health, nutrition and treatment of common childhood diseases like diarrhea, pneumonia, malaria and severe malnutrition) given by health extension workers. In line with this, the households must also implement at least 75% and above of the packages listed above; and certified by districts health office for their achievement. Then, when respondents comply with the above conditions, it was scored in to yes otherwise into no.

Pregnancy complication was measured based the following medical conditions during pregnancy period: vaginal bleeding, abdominal pain, persistence of back pain, blurry vision, no fetal movement and swelling of hands or face. Delivery complication was measured based on the following medical conditions during delivery time: mal presentation, obstructed labor, meconium stained amniotic fluid, premature rapture of membrane (< 1 days), umbilical cord prolapsed, vaginal bleeding and retained placenta (> 30 min). For both delivery and pregnancy complication, the above medical conditions were predefined in the questionnaires. So mothers were responded from the predefined medical conditions; if they answered presence of at least one or more signs from the list, it was scored yes unless scored no.

Neonate size at birth was proxy indicator of birth weight at birth and measured by perception of the mother. It was labeled in to small and average size neonate. Small size neonates was proxy indicator of neonates with low birth weight (< 2500 g) and average size neonates was proxy indicator of neonates with normal birth weight (2500–4200 g). Early initiation of breast-feeding was measured when the neonates start breast-feeding with in 1 h (= < 1 h) after birth and late initiation of breast-feeding when the neonates start breast-feeding > = 2 h after birth. Birth attendant was defined when mothers were assisted by health professionals (physicians, nurses, health officers, and health extensions trained in clean delivery) during delivery time, then it was labeled into skilled birth attendant otherwise labeled in to unskilled birth attendant. Structured interviewer administered questionnaire was used to collect information from each participants. Interview for the mothers were conducted face to face. Twelve diploma nurses, currently working on maternal and child health in health center and four bachelor sciences nurses currently working as health center –health post linkage focal person in health center; who speak and understand local language were recruited and trained as data collectors and supervisors respectively. Before data collection, the instrument was pretested in 5% of the total sample size. During data collection, the administered questionnaires were checked for completeness and consistency on daily basis by supervisors. After data collection completed, the principal investigator checked the data during data entry, cleaning, and analysis.

### Analysis

Data was entered, processed, and analyzed using EPI Info version 7 and SPSS version 16 softwares (Additional file 1). Descriptive analysis was done, to check for outliers and inconsistencies. Then, univariate conditional logistic regression analysis was performed to identify candidates for multivariate conditional logistic regression. Strength of the association was measured using parameters of crude matched odd ratio (CmOR) with 95% confidence interval (CI) and significance test at *P* value < 0.05. An inclusion criterion for multivariate conditional logistic regression was P -value < 0.2. Backward elimination strategy was used to build the final model and measure of association of each predictors were determined using parameter of adjusted matched odds ratio (AmOR) with 95%CI. Statistical significance was tested using Wald statistical test at P value < 0.05. In line with this, biologically meaningful interactions were assessed for inclusion in the final model. Furthermore, multicollinearity was checked using variance inflation factor (VIF) parameter, with acceptable range of 1–10 coefficients. Accordingly, collinearity was found between parity and gravidity; and diagnostic was made by removing gravidity from the model. Finally, goodness of fit of the final model was tested using likelihood ratio statistics and it was found fit.

## Results

The preliminary data revealed that, from September 1, 2010 until September 1, 2013, 2075 neonates were born alive. Out of those live birth (LB) neonates, 139 singleton neonates were dead during the first 28 completed days after birth. As a result, NMR was 66.9 per 1000 LB. Totally 114 mothers of cases and 114 mothers of controls were included in the study, with 95.8% response rate. Among 114 cases, 27(23.68%), 44(38.6%), 43(37.72%) died within first day, 2–7 days, 8–28 days respectively. The mean age of mothers of cases and controls were 30.3(±6.85) and 30.1(±4.89) respectively. The median household income was 800 birr for cases and 900 birr for controls. One hundred three mothers of cases (90.35%) and 106 (92.98%) mothers of controls were married. With regard to ethnicity, 84(73.68%) mothers of cases, 82(71.93%) mothers of control were Berta, and 24(21.05%) mothers of cases and 25(21.93%) mothers of control were Amhara. With regard to religion, ninety-one mothers of cases (79.82%) and 90(78.95%) mothers of controls were Muslim. Ninety-one mothers of cases (79.82%) and seventy mothers of controls were illiterate (61.40%) (Table 1).

**Table 1:** Univariate association of socio demographic and socioeconomic factors and neonatal mortality in Assosa zone, Western Ethiopia, 2013

	Case(*n* = 114)	Control(n = 114)		
Variables	number (%)	number (%)	CmOR(95%CI)	*P*-value
Age of the mother				
< 20	2(1.75)	2(1.75)	1.47(0.19–11.11)	0.707
20–34	70(61.40)	87(76.32)	1	
> =35	42(36.84)	25(21.93)	2.17(1.16–4.04)	0.014*
Marital status				
Married	103(90.35)	106(92.98)	1	
Widowed / Divorced	11(9.65)	8(7.02)	1.42(0.54–3.75)	0.469
Ethnicity				
Berta	84(73.68)	82(71.93)	1.17(0.39–3.39)	0.769
Amhara	24(21.05)	25(21.93)	1.10(0.32–3.74)	0.871
Others	6(5.26)	7(6.14)	1	
Religion				
Muslim	91(79.82)	90(78.95)	1.05(0.55–2.01)	0.869
Christian	23(20.18)	24(21.05)	1	
Educational status				
Illiterate	91(79.82)	70(61.40)	2.5(1.34–4.64)	0.004*
Literate	23(20.18)	44(38.60)	1	
Occupation				
House wife	48(42.11)	58(50.88)	0.96(0.38–2.43)	0.945
Gold miner	35(30.70)	24(21.05)	1.62(0.58–4.48)	0.351
Farmer	21(18.42)	21(18.42)	1.04(0.32–3.33)	0.938
Others	10(8.77)	11(9.65)	1	
Family size				
*< 5*	51(44.74)	69(60.53)	1	
*> =5*	63(55.26)	45(39.47)	1.90(1.10–3.26)	0.020*
Monthly income				
< 608 birr	32(28.07)	21(18.42)	1.68(0.90–3.13)	0.097
> =608 birr	82(71.93)	93(81.58)	1	
No of rooms				
< =2	98(85.96)	101(88.60)	0.76(0.33–1.75)	0.533
> =3	16(14.04)	13(11.6)	1	
Roof of the house				
Corrugated iron sheet	31(27.19)	26(22.81)	1	
Thatched	83(72.81)	88(77.19)	0.76(0.39–1.46)	0.412
Separate kitchen				
Yes	51(44.74)	48(42.11)	1.12(0.65–1.89)	0.686
No	63(55.26)	66(57.89)	1	
Water source				
Safe	92(80.70)	100(87.72)	1	
Unsafe	22(19.30)	14(12.28)	1.61(0.80–3.22)	0.174
Toilet facility				
Yes	82(71.93)	92(80.7)	0.62(0.34–1.15)	0.135
No	32(28.07)	22(19.3)	1	
Model HHs in HEP				
Yes	35(30.70)	67(58.77)	0.33(0.18–0.58)	< 0.001*
NO	79(69.30)	47(41.23)	1	

*variables statistical significant at p value < 0.05

*CmOR*: Crude matched Odds Ratio, *HHs*: House Holds, *HEP* – Health Extension package

During univariate analysis of socio demographic factors, Age of the mother > = 35 years [CmOR = 2.17; 95%CI: 1.16–4.04], illiterate mothers [CmOR = 2.5; 95%CI: 1.34–4.64], family size > = 5[CmOR = 1.19; 95%CI: 1.10–3.26], and model households (HHs) in health extension package (HEP) [CmOR = 0.33; 95%CI: 0.18–0.58] were found associated with NM at 5% level of significance. However, marital status, ethnicity, religion, occupation, type of roof of the house, presence of separate kitchen, type of water source, and presence of toilet facility were not significantly associated with NM (Table 1).

With regard to maternal biological and obstetric factors, age at first pregnancy < 20 years old [CmOR = 8.4; 95%CI: 3.32–21.23], gravidity of one [CmOR = 4.31; 95%CI: 2.14–8.65] and gravidity of five and more [CmOR = 4.10; 95%CI: 1.77–9.47], parity of one [CmOR = 4.21; 95%CI: 2.17–8.14] and parity of five and more [CmOR = 3.99; 95%CI: 1.52–10.44], previous history of still birth [CmOR = 3.00; 95%CI: 1.09–8.24]), ANC visit [CmOR = 0.30; 95%CI: 0.17–0.52], pregnancy complication ([CmOR = 3.00; 95%CI: 1.67–5.38],delivery complication [CmOR = 3.57; 95%CI: 1.04–6.45], and birth interval with previous birth < 24 months [CmOR = 2.66; 95%CI: 1.04–6.81] were statistically significantly associated in the univariate analysis. Whereas, previous history of NM, previous history of abortion, mode of delivery, and tetanus toxoid (TT) vaccination were not statistically significantly associated (Table 2)**.**

**Table 2:** Univariate association of maternal biological and obstetric factors and neonatal mortality, in Assosa zone, Western Ethiopia, 2013

Variables	Case(n = 114)	Control(n = 114)	CmOR(95%CI)	*P*-value
	number(%)	number(%)		
Delivery complication				
Yes	70(61.40)	34(29.82)	3.57(1.97–6.45)	0.004*
No	44(38.0)	80(70.18)	1	
Age at first pregnancy				
< 20	46(40.35)	9(7.89)	8.40(3.32–21.23)	< 0.001*
> =20	68(59.65)	105(92.11)	1	
Parity				
I	59(51.75)	28(24.56)	4.21(2.17–8.14)	< 0.001*
II-IV	38(33.33)	79(69.30)	1	
V+	18(18.42)	7(6.14)	3.99(1.52–10.44)	0.004*
Gravidity				
I	51(44.74)	24(21.05)	4.31(2.14–8.65)	< 0.001*
II-IV	39(34.21)	81(71.05)	1	
V+	24(21.05)	9(7.89)	4.10(1.77–9.47)	0.001*
Birth interval with previous birth				
< 24 months	50(84.75)	45(38.46)	2.66(1.04–6.81)	0.033*
> =24 month	9(15.25)	44(49.44)	1	
Previous history of still birth				
Yes	16(14.04)	6(5.26)	3.00(1.09–8.24)	0.025*
No	98(85.96)	108(94.74)	1	
Previous history of abortion				
Yes	1(0.88)	2(1.75)	0.50(0.05–5.51)	0.571
No	113(99.12)	112(98.25)	1	
Previous history of NM				
Yes	13(11.40)	5(4.39)	3.00(0.97–9.29)	0.0578
No	101(88.6)	109(95.61)	1	
ANC visit				
Yes	47(41.23)	84(73.68)	0.30(0.17–0.52)	< 0.001*
No	67(58.77)	30(26.32)	1	
TT dose received				
< 2	9(7.89)	5(4.39)	3.00(0.60–14.60)	0.178
2 to5	105(92.11)	109(95.61)	1	
Pregnancy complication				
Yes	56(49.12)	26(22.81)	3.00(1.67–5.38)	< 0.001*
No	58(50.88)	88(77.19)	1	

*Variables statistically significant at *P* value < 0.05, *ANC*: antenatal care, *TT*: tetanus toxoid, *NM*: neonatal mortality

Neonatal factors, like small size neonate at birth [CmOR = 5.00; 95%CI: 2.44–10.22], gestational age < 37 weeks [CmOR = 5.42; 95%CI: 2.42–12.15], partial breast-feeding [CmOR = 3.55; 95%CI: 1.69–7.44], and vaccination at birth [CmOR = 0.35; 95%CI: 1.19–0.66] were statistically significantly associated with NM in univariate analysis. Nevertheless, sex of the neonates and initiation time of breast-feeding were not statistically significantly associated (Table 3)**.**

**Table 3:** Univariate association of neonatal related factors and neonatal mortality in Assosa zone, Western Ethiopia, 2013

	Case(n = 114)	Control(n = 114)		
Variables	number(%)	number(%)	CmOR(95%CI)	*P* value
Sex of the neonate				
Male	57(50)	56(49.12)	1.03(0.66–1.75)	0.892
Female	57(50)	58(50.88)	1	
Size of neonates at birth				
Average size	61(53.51)	97(85.09)	1	
Small size	53(46.49)	17(14.91)	5.00(2.44–10.22)	< 0.001*
Gestational age				
> =37 weeks	70(61.40)	101(88.60)	1	
< 37 weeks	44(38.60)	13(11.40)	5.42(2.42–12.15)	< 0.001*
Time of breast feeding initiation				
< =1 h (early)	19(21.84)	25(21.93)	1	
> =2–24 h (late)	68(78.16)	89(78.07)	1.13(0.56–2.26)	0.723
Breast feeding practice				
Exclusive	14(16.09)	51(44.74)	1	
Partial	73(83.91)	63(55.26)	3.55(1.69–7.44)	< 0.001*
Vaccination at birth				
Yes	32(28.07)	57(50)	0.35(0.19–0.66)	0.001*
No	82(71.93)	57(50)	1	

*Variables statistically significant at P- value < 0.05

Concerning health service and delivery factors, home delivery [CmOR = 2.30; 95%CI: 1.36–3.88], postnatal care (PNC) [CmOR = 0.13; 95%CI: 0.05–0.31] and unskilled birth attendant [CmOR = 3.4; 95%CI: 1.25–9.21] were statistically significantly associated with NM. Despite of the above, distance of health facility from home were not shown statistically significant association (Table 4)**.**

**Table 4:** Univariate association of delivery and health service related factors and neonatal mortality in Assosa zone, Western Ethiopia, 2013

	Case(*n* = 114)	Control(*n* = 114)		
Variables	number(%)	number(%)	CmOR(95%CI)	*P*- value
Place of delivery				
Home	76(66.67)	50(43.86)	2.3(1.36–3.88)	0.001*
HF	38(33.33)	64(56.14)	1	
Distance of HF from home				
< =2 h	105(92.11)	108(94.74)	1	
> 2 h	9(7.89)	6(5.26)	1.5(0.53–4.21)	0.443
Delivery attendants				
Skilled	31(40.79)	37(74)	1	
Unskilled	45(59.21)	13(26)	3.40(1.25–9.21)	0.016*
Mode of delivery				
Vaginal	103(90.35)	107(93.86)	1	
C-section	11(9.65)	7(6.14)	1.5(0.53–4.21)	0.441
PNC visit				
Yes	64(56.14)	102(89.47)	0.13(0.05–0.31)	< 0.001*
No	50(43.86)	12(10.53)	1	

*Variables statistically significant at P value < 0.05 PNC: Post Natal Care, HF: Health Facility

From behavioral and psychosocial factors, only heavy work exercise during pregnancy [CmOR = 2.41; 95%CI: 1.37–4.24] was statistically significantly associated with NM in the univariate analysis. On the other hand, pregnancy planning, reaction of family to pregnancy, and domestic violence during pregnancy were not statistically significantly associated (Table 5).

**Table 5:** Univariate association of behavioral and psychosocial related factors and neonatal mortality in Assosa zone, Western Ethiopia, 2013

	Case(n = 114)	Control(n = 114)		
Variables	number (%)	number (%)	CmOR(95%CI)	*P* value
Pregnancy planning				
Planned	65(57.02)	79(69.30)	0.61(0.35–1.03)	0.068
Un Planned	49(42.98)	35(30.70)	1	
Reaction of family to pregnancy				
Happy	103(90.35)	106(92.98)	1	
Not happy	11(9.65)	8(7.02)	1.37(0.55–3.41)	0.493
Domestic violence during pregnancy				
Yes	13(11.40)	11(9.65)	1.18(0.52–2.63)	0.683
No	101(88.60)	103(90.35)	1	
Heavy work exercise during pregnancy				
Yes	54(47.37)	30(26.32)	2.41(1.37–4.24)	0.002*
No	60(52.3)	84(73.68)	1	

*Variables statistically significant at P -value < 0.05

In the multivariate conditional logistic regression analysis, model HHs in HEP, age at first pregnancy < 20 years old, pregnancy complication, delivery complication, antenatal care visit, parity of one (primipara mothers), small size neonates at birth, gestational age < 37 weeks, and home delivery were found statistically significantly associated with NM (Table 6).

**Table 6:** Multivariate analysis of selected explanatory factors and neonatal mortality in Assosa zone, Western Ethiopia, 2013

Variables	AmOR(95%CI)	*P*- value
Age of the mother		
< 20	0.14 (0.01–2.22)	0.161
20–34	1	
> =35	1.87(0.72–4.85)	0.193
Educational status		
Illiterate	2.59(0.87–7.65)	0.553
Literate	1	
Family size		
*< 5*	1	
*> =5*	1.53(0.64–3.65)	0.335
Monthly income		
< 608 birr	0.45(0.18–1.09)	0.078
> =608 birr	1	
Model HHs in HEP		
Yes	0.32(0.12–0.86)	0.024*
No	1	
Water type		
Safe	1	
Unsafe	1.00(0.37–2.67)	0.997
Toilet Facility		
Yes	0.75(0.32–1.75)	0.518
No	1	
Age at 1st pregnancy		
< 20	4.30(1.13–16.27)	0.031*
> =20	1	
Pregnancy complication		
Yes	4.59(1.53–13.78)	0.006*
No	1	
Delivery complication		
Yes	2.80(1.06–7.39)	0.037*
No	1	
Parity		
I	3.37(1.05–10.78)	0.041*
II-IV	1	
V+	1.67(0.34–8.38)	0.527
Birth interval with previous birth		
< 24 months	4.02 (0.59–27.42)	0.154
> =24 month	1	
Previous history of still birth		
Yes	3.4 (0.50–23.86)	0.203
No	1	
Previous history of NM		
Yes	6.9 (0.34–13.96)	0.206
No	1	
ANC visit		
Yes	0.34 (0.12–0.94)	0.038*
No	1	
TT dose received		
< 2	1	
2–5	0.25(0.01–13.43)	0.498
Size of neonates at birth		
Average	1	
Small	3.40(1.05–11.55)	0.049*
Type of breast feeding practice		
Exclusive	1	
Partial	2.06(0.14–13.23)	0.597
Vaccination at birth		
Yes	0.56(0.16–2.09)	0.382
No	1	
Gestational age		
< 37-Week	4.35(1.16–16–28)	0.029*
> =37 Week	1	
Place of delivery		
Home	2.84(1.07–7.55)	0.035*
HF		
Delivery attendants		
Skilled	1	
Unskilled	2.00(0.79–5.02)	0.139
PNC visit		
Yes	0.31(0.04–2.68)	0.291
No	1	
Heavy work exercise during pregnancy		
Yes	2.35(0.93–5.92)	0.074
No	1	
Pregnancy planning		
Planned	0.67(0.27–1.66)	0.339
Unplanned	1	

*Independent predictor variables, statistically significant at P -value < 0.05

Risk of NM was 68% lower [AmOR = 0.32; 95% CI: 0.12–0.86] in model HHs of HEP compared with those not model HHs in HEP.

Neonates born to mother’s age at 1st pregnancy < 20 years old were 4.3 times at higher risk of death [AmOR = 4.3; 95% CI: 1.13–16.27] than those neonates born to mother’s age at first pregnancy > = 20. Neonates born to mothers who had complication during pregnancy were 4.59 times more likely at risk of death [AmOR =4.59; 95% CI: 1.53–13.78] compared with those neonates born to mothers who had no complication during pregnancy. Mothers who had complication during delivery were 2.8 fold at higher risk of having NM [AmOR = 2.80; 95% CI: 1.06–7.39] compared with those mothers who had no complication. Primipara mothers were 3.37 fold at higher risk of having neonatal death [AmOR =3.37; 95% CI: 1.05–10.78] than those mothers with parity of two to four. Neonates born to mothers who had received ANC were 66% lower risk of death [AmOR = 0.34; 95% CI: 0.12–0.94] compared with those neonates born to mothers who had not received ANC.

Neonates who were small size at birth were 3.4 times more likely at risk of death [AmOR = 3.40; 95% CI: 1.05–11.55] compared with those average size neonates at birth. In line with this, neonatal death was 4.35 times higher among neonates with gestational age (GA) < 37 weeks [AmOR = 4.35; 95%CI: 1.16–16.28] compared with those neonates with GA > =37 weeks. Home delivered mothers were 2.8 times at higher risk of having neonatal death [AmOR = 2.84; 95% CI: 1.07–7.55] than those health institution delivered mothers.

## Discussion

This study was conducted to identify predictors of neonatal mortality. It was employed community based matched case control design. This study revealed that, model HHs in HEP, age at first pregnancy < 20 years old, pregnancy and delivery complication, ANC visit, parity of one (primipara mothers), small size neonates at birth, GA < 37 weeks, and home delivery were found to be predictors of NM.

Model HHs in HEP were significantly associated with neonatal mortality. Even though, there were no previous studies done specifically on association of model HHs in HEP and NM; previous reports by United Nations International Children’s Fund and World Bank were indicated model HHs in HEP was major factor in decreasing child mortality in Ethiopia [14, 15]. This might be explained due to the fact that, model household families have better health awareness and practices through implementing the 16 health packages, that are directly and indirectly related with maternal and child health services. The packages comprise broad health services that can be implemented at home or health facilities, through improving health-seeking behavior of the mother during antenatal and postnatal periods. Thus, improvement of the health of the mothers and better caring for their newborn neonates might have significant effect for decreasing the risk of NM. On the other hand, maternal education, household income, and family size were not significantly associated with NM. This finding was similar with study finding from Ghana [13], but not consistent with studies done in Vietnam and Brazil [19, 20]. This discrepancy might be probably because of small sample size of this study to detect small effect size measure. However, educational status of the mothers and income of the households could be clinically significantly associated.

Concerning maternal biological and obstetric factors, mother’s age < 20 years old at first pregnancy was shown statistically significant association with NM. This finding was congruent with previous studies [17, 18, 20]. This could be explained due to the fact that, early age pregnant mothers might not physically and psychologically ready to have child; have less awareness and knowledge on pregnancy and new born care. Hence, those all could have negative effect on health seeking behavior of the mother for ANC and PNC services. Consequently, this might made them susceptible to have high risk of pregnancy and delivery complications, risk of having preterm birth, and LBW neonates; which are known risk factors for NM. Pregnancy and delivery complication were significantly associated with NM. This finding was supported by previous studies [17, 21–24]. This is a known fact that, neonates born to mothers who had pregnancy complications were at higher risk of having neonatal death because mothers might be exposed to higher risk of nutritional problems, having high probability of preterm birth, and LBW neonates. In line with this, mothers who have delivery complication could have high probability of risk of neonatal death, due to the fact neonates may have high risk of having sepsis, birth asphyxia, congenital malformation, and birth trauma, which are known cause of NM. Primipara mothers had shown higher risk of having neonatal death. This finding was confirmed by findings of previous studies [13, 21, 23]. This could be explained due to the fact that, primipara mothers might have poor knowledge and skills about newborn care (such as exclusive breast-feeding practice, early initiation of breast-feeding and kangaroo care for new born). In line with this, they may have poor awareness to seek medical care during antenatal and postnatal period. Neonates born to mothers who had received ANC were statistically significantly associated with decreasing risk of neonatal death. This finding was in agreement with findings of previous studies done in Egypt, Vietnam, Kenya, and Brazil [18, 19, 21, 24]. This was probably receiving ANC could improve nutritional status of the mother, receiving iron and folic acid, TT immunization, and early detection of complications during pregnancy, consequently those could contribute to have low risk of NM. However, birth space with previous birth < 24 months, previous history of stillbirth, previous history of NM, and TT vaccination were not statistically significantly associated. This finding was not consistent with previous studies [18, 24, 25]. This disagreement might be due to homogeneous distribution of the risk factors (previous history of stillbirth, previous history of NM, and TT vaccination) in both comparable groups, as well as small sample size of the study. Despite of the above non-statistical significance, birth space with previous birth < 24 month could be clinically significantly associated.

Among the neonatal related factors, small size neonates were at higher risk of death than those average size neonates were. This finding was similar with previous studies conducted in Kenya, Ethiopia, Nigeria, and Indonesia [21, 26–28]. This might be small size neonates were highly susceptible for different infections due to having low immunity defense. Neonatal death was statistically higher among neonates with GA < 37 weeks. This result was in line with results of previous studies [21, 24]. This is a well-known fact that, neonates born with GA < 37 weeks are exposed to many physiologic challenges and fatal conditions to adapt the extra uterine life; such as hypothermia, respiratory distress, different cardio vascular and hematological conditions, like anemia and hyperbilirubinemia. In line with this, immature immune defense can also expose them to infections, nutritional deficiencies, gastro intestinal problems, and electrolyte imbalance. Nevertheless, breastfeeding practice and vaccination at birth were not shown significant association. This finding was similar with previous study from Kenya [26], however, study from Indonesia was revealed significant association [28]. Vaccination at birth was not statistically significantly associated; vaccination at birth might not have immediate clinical significance during the neonatal period. On the other hand, majority of the neonates in both comparable groups were partial breast-fed, this could lead to statistically non-significance association. However, clinically this could be significantly associated.

Concerning health service and delivery related factors; neonatal death was statistically significantly higher among neonates born to home delivered mothers. This finding was in line with findings from previous studies done in Ethiopia and Vietnam [33, 34]. Neonates born to home delivered mothers are highly exposed to different infections and birth traumas, due to absence of safe and clean delivery kits, professional birth attendant, and essential newborn care practices especially during the first hour immediately after birth;this could increase risk of death. However, mode of delivery, PNC, and unskilled birth attendant were not statistically significantly associated with NM. Finding of this study about mode of delivery was similar with findings of previous studies [29, 30], but finding about unskilled birth attendant and PNC were not consistent with previous study [16]. This disagreement might be due small sample size of this study to detect small effect size measure of both explanatory factors. In addition, distribution of type of birth attendant was homogenous in both comparable groups. Although both factors were not statistically significant in this study, clinically could be significantly associated.

Concerning physical and psychological factors, heavy work exercise and pregnancy planning were not statistically significantly associated with risk of neonatal death. Conversely, previous studies reported statistically significant association [35, 36]. This discrepancies could be due to mothers who were reported they had heavy work exercise and violence during pregnancy period were very few and similar distribution in both comparable groups.

Generally, based on the findings of this study, EDHS report, and general situation of the region; there were three suggested reasons for increasing NMR in the region, while the national NMR was decreased between 2005 and 2011. Firstly, health services coverage of the region (model HHs in HEP, ANC, PNC, and institutional delivery) were very low compared with health services coverage of the rest regions in the country. Secondly, poor access and utilization of basic emergency obstetric care, and intensive newborn care centers in the region. The third reasons were, early marriage and early pregnancy, and home delivery were wide spread cultural practice in the region. Even though, the rate of NM of the region was increased between 2005 and 2011, it had no effect on the national NMR for the same period; due to small population proportion of the region compared with other regions in country.

### Study strengths and limitations

At last, it is crucial to discuss strengths as well as limitations of this study. The study employed matched case control design, subsequently this could improve efficiency to control cofounders by increasing precision of effect size estimate. Moreover, this study was community based; hence, it gave an opportunity to investigate risk factors present within the community.

Despite the above strengths, the study also had limitations. Firstly, majority of the data was three-year recall period self reported data by mothers. Hence, this might introduced recall bias due to differential recall of information among mothers of cases and mothers of controls; this may lead to differential misclassification of study subjects and finally distortion of effect size estimates in any direction from null. Secondly, majority of the socio demographic factors were temporal measurements but taken as proxy indicators of the past and this might not give true measurement of the past. The third limitation was, small size and average size neonates was proxy indicator of birth weight of neonates, due to absence of birth weight record and this might not be accurate measurement. Lastly, nutritional status of the mothers and delivery related factors (type of cord cutting and tie materials, delivery surfaces) were not studied in detail. Therefore, the reader of this article should consider those limitations might have implication on internal validity of the result.

### Clinical implications

Based on the findings of this study, improving coverage of model HHs in HEP will have broad impact to reduce neonatal mortality, since it comprise many health packages directly and indirectly related to maternal and new born health, that are crucial to implement at family level. Secondly, improving access and utilization of basic emergency obstetric and intensive newborn care service to reduce risk of delivery complications and improve newborn survival especially for preterm and LBW neonates respectively. The third interventions are promotion of ANC, institutional delivery, health education and family planning for adolescents to prevent early age pregnancy. Generally, those interventions should be implemented at community level to avert the high neonatal mortality in the region.

### Future research

Nutritional status of the mother and delivery related risk factors, such as: cord cutting material, delivery surface, and cord tie material used during delivery were not investigated in detail and should be studied in future.

## Conclusions

In summary, model households in health extension packages, mothers age at first pregnancy < 20 years old, complication during pregnancy, complication during delivery, primipara mothers, antenatal care visit, small size neonates at birth, gestational age < 37 weeks, and home delivery were found as independent predictors of neonatal death. Therefore, promotion of model HH in HEPs, ANC visit, institutional delivery, and prevention of early age pregnancy and improve access of basic emergency obstetric care and intensive newborn care are very crucial interventions to avert the high risk of neonatal mortality.

## Additional files

Additional file 1: Anonymous data generated and analyzed to study predictors of neonatal mortality in Assosa Zone, Western Ethiopia, 2013. (XLSX 99 kb)

## Abbreviations

AmOR: Adjusted matched Odd Ratio
ANC: Anti Natal Care
CI: Confidence Interval
CmOR: Crude matched Odd Ratio
EDHS: Ethiopian Demographic Health Survey
GA: Gestational Age
HEP: Health Extension Package
HF: Health Facility
HHs: Households
LBW: Low Birth Weight
MDG: Millennium Development Goal
NM: Neonatal Mortality
NMR: Neonatal Mortality Rate
PNC: Post Natal Care
SPSS: Statistical Package for Social Science
TT: Tetanus Toxoid
VIF: Variance Inflation Factor
WHO: World Health Organization
